# First in patient assessment of brain tumor infiltrative margins using simultaneous time-resolved measurements of 5-ALA-induced PpIX fluorescence and tissue autofluorescence

**DOI:** 10.1117/1.JBO.27.2.020501

**Published:** 2022-02-02

**Authors:** Alba Alfonso-García, Xiangnan Zhou, Julien Bec, Silvia N. Anbunesan, Farzad Fereidouni, Lee-Way Jin, Han S. Lee, Orin Bloch, Laura Marcu

**Affiliations:** a University of California Davis, Department of Biomedical Engineering, Davis, California, United States; b University of California Davis, Department of Pathology and Laboratory Medicine, Sacramento, California, United States; c University of California Davis, Department of Neurological Surgery, Sacramento, California, United States

**Keywords:** fluorescence lifetime imaging, 5-ALA-induced PpIX fluorescence, nicotinamide adenine (phosphate) dinucleotide, brain tumor, glioblastoma

## Abstract

**Significance:**

5-aminolevulinic acid (5-ALA)-induced protoporphyrin IX (PpIX) fluorescence is currently used for image-guided glioma resection. Typically, this widefield imaging method highlights the bulk of high-grade gliomas, but it underperforms at the infiltrating edge where PpIX fluorescence is not visible to the eyes. Fluorescence lifetime imaging (FLIm) has the potential to detect PpIX fluorescence below the visible detection threshold. Moreover, simultaneous acquisition of time-resolved nicotinamide adenine (phosphate) dinucleotide [NAD(P)H] fluorescence may provide metabolic information from the tumor environment to further improve overall tumor detection.

**Aim:**

We investigate the ability of pulse sampling, fiber-based FLIm to simultaneously image PpIX and NAD(P)H fluorescence of glioma infiltrative margins in patients.

**Approach:**

A mesoscopic fiber-based point-scanning FLIm device (355 nm pulses) was used to simultaneously resolve the fluorescence decay of PpIX (629/53 nm) and NAD(P)H (470/28 nm). The FLIm device enabled data acquisition at room light and rapid (<33  ms) augmentation of FLIm parameters on the surgical field-of-view. FLIm measurements from superficial tumors and tissue areas around the resection margins were performed on three glioblastoma patients *in vivo* following inspection of PpIX visible fluorescence with a conventional neurosurgical microscope. Microbiopsies were collected from FLIm imaged areas for histopathological evaluation.

**Results:**

The average lifetime from PpIX and NAD(P)H fluorescence distinguished between tumor and surrounding tissue. FLIm measurements of resection margins presented a range of PpIX and NAD(P)H lifetime values ($\tau_{\mathrm{PpIX}\;}∼3$ to 14 ns, $\tau_{\mathrm{NAD}(P)H}=3$ to 6 ns) associated with unaffected tissue and areas of low-density tumor infiltration.

**Conclusions:**

Intraoperative FLIm could simultaneously detect the emission of PpIX and NAD(P)H from patients *in vivo* during craniotomy procedures. This approach doubles as a clinical tool to identify tumor areas while performing tissue resection and as a research tool to study tumor microenvironmental changes *in vivo*. Intraoperative FLIm of 5-ALA-induced PpIX and tissue autofluorescence makes a promising surgical adjunct to guide tumor resection surgery.

## 1 Introduction

Fluorescence-guided surgery (FGS) for primary brain tumor (glioma) resection using 5-aminolevulinic acid (5-ALA) improves the extent of tumor resection in high-grade glioma patients, directly impacting patient survival. Its use is a widespread practice in Europe and is rapidly gaining popularity in the United States.^1^ Oral administration of 5-ALA results in selective accumulation of protoporphyrin IX (PpIX) in tumor cells.^2^ In glioblastoma (GBM) patients, PpIX fluorescence (emission peak at 635 nm upon 405 nm excitation^3^) is strong enough for visual assessment using a surgical microscope, and it enables guided tumor resection. However, for the low-cellularity infiltrating edge of high-grade gliomas and most low-grade gliomas, the PpIX fluorescence intensity decreases below the visible threshold.^4^ Current implementations of PpIX fluorescence visualization systems require working in a dark surgical field, hindering simultaneous tumor identification and resection. Additionally, visible PpIX fluorescence suffers from non-specific intensity variations (e.g., non-uniform illumination) that complicate the quantitative assessment of tumor with high accuracy.^5^ Recent studies conducted on excised brain tissue have suggested that fluorescence spectroscopy and lifetime imaging enable further quantification of PpIX fluorescence below the visible detection threshold.^6^^,^^7^ Time-resolved fluorescence is expected to increase the detection sensitivity of areas with weak fluorescence intensity because fluorescence lifetime is largely independent of tissue absorption and scattering properties, and unlike intensity alone, it is robust to variations in tissue-detector distance.

PpIX has a long fluorescence lifetime that is also present when excited with near-UV.^8^ Therefore, using 355 nm excitation instead of 405 nm, it is possible to simultaneously excite both PpIX and energy metabolic cofactor nicotinamide adenine (phosphate) dinucleotide [NAD(P)H]. The spectral and lifetime properties of NAD(P)H have been used to infer metabolic changes in tumor cells and tissues during surgery.^9^^–^^11^ In glioma tissue, NAD(P)H levels vary according to isocitrate dehydrogenase (IDH) mutation status^12^ and influence 5-ALA generation and PpIX accumulation.^13^ The ability to measure both PpIX and NAD(P)H fluorescence could enable studies that investigate variations in PpIX accumulation for different tumor types upon 5-ALA uptake.^14^ The addition of NAD(P)H fluorescence offers the possibility to study tissue properties (e.g., metabolism and necrosis) that could further aid in the guidance of tumor resection. ^9^^,^^15^ Although a recent study conducted on excised brain tissue specimens demonstrated improved classification accuracy in the infiltration zone and low-grade gliomas when NAD(P)H lifetime was combined with PpIX lifetime,^16^ no study in patients (*in vivo*) was reported. This proof-of-concept study aims to demonstrate the use in patients of pulse sampling fluorescence lifetime imaging (FLIm) with a single excitation source at 355 nm to simultaneously detect PpIX and NAD(P)H time-resolved fluorescence of glioma infiltrative margins.

## 2 Methods

FLIm was performed on three GBM patients who underwent standard craniotomies for tumor resection under 5-ALA guidance. Data were acquired with a custom-made intraoperative mesoscopic FLIm device.^17^ This study was approved by the University of California, Davis Institutional Review Board. Informed consent was obtained from each patient.

Patients with newly diagnosed GBM were given a single oral dose of 5-ALA (Gleolan 20 mg/kg, NX Development Corp, Lexington KY) 3 h prior to induction of anesthesia. During routine craniotomy for tumor resection, PpIX fluorescence was visualized with the surgical microscope (OPMI Pentero 900, Carl Zeiss AG, Germany) with a 405 nm excitation and an emission filter of 665/90 nm. FLIm measurements were acquired from the cortical surface prior to surgical resection and from resection margins (five per patient) around the surgical cavity after removal of the majority of the tumor. Areas with and without visible PpIX fluorescence were selected for FLIm measurement and biopsy. Each area was first imaged under 405 nm illumination to visualize the PpIX fluorescence with the standard surgical microscope (deemed positive or negative by the primary surgeon). Next, under white-light illumination, the area was scanned with the fiber optic probe for FLIm measurements. The measured area was cleared of blood and fluids prior to data acquisition. Finally, micro-biopsies (1 to $2\;{\mathrm{mm}}^{3}$) were collected from the FLIm scanned locations for histopathological evaluation. Tissue was formalin-fixed and paraffin-embedded for analysis of hematoxylin and eosin (H&E) stained sections to provide the final diagnosis and determine the tumor cellularity (absent, low, and high) of each sample. Immunohistochemistry for the cell proliferation marker Ki67 was performed to further verify the presence of tumor in low cellularity regions. Histopathologic sections were all analyzed and graded by a single board-certified neuropathologist (H.S.L.).

The FLIm device relied on a time-domain pulse sampling approach to resolve fluorescence temporal dynamics excited by a 355 nm pulsed laser (480 Hz, 0.25 μJ pulse energy, and 600 ps pulse duration).^17^ Multispectral detection of tissue fluorescence over three spectral bands (390/40, 470/28, and 629/53 nm) was facilitated by three avalanche photodiodes (APD) (32.9, 64.5, and 93.2 kV/W conversion gain at 390, 470, and 629 nm, respectively). These APD detectors present a higher quantum efficiency (>80% at 629 nm) than the microchannel plate photomultiplier tube used in previously reported FLIm devices (<10% at 629 nm).^9^ The analysis presented here focused on fluorescence detected at channels 470/28 and 629/53 nm, and for simplicity, we refer to them as NAD(P)H and PpIX channels, respectively. An additional laser at 445 nm was used to highlight the measurement location to enable real-time (video rate) augmentation of the fluorescence metrics onto the neurosurgical microscope video stream.^18^

The average fluorescence lifetime ($\tau_{\mathrm{avg}}$) of the brain tissue was extracted from the measured fluorescence decays with a non-negative least-squares deconvolution with Laguerre expansion in each spectral band,^19^ and it is reported as the mean and standard deviation of each region of interest (ROI) and margin image as indicated in the Results and Discussion section. The phasor approach^20^ was employed to visualize the lifetime dynamics. The 2D coordinates of the phasor plot consisted of the real (Re) and imaginary (Im) parts of the Fourier transform of the intensity normalized fluorescence decays.

## 3 Results and Discussion

### 3.1 Glioma Infiltrating the Cortex

**Fig. 1:**
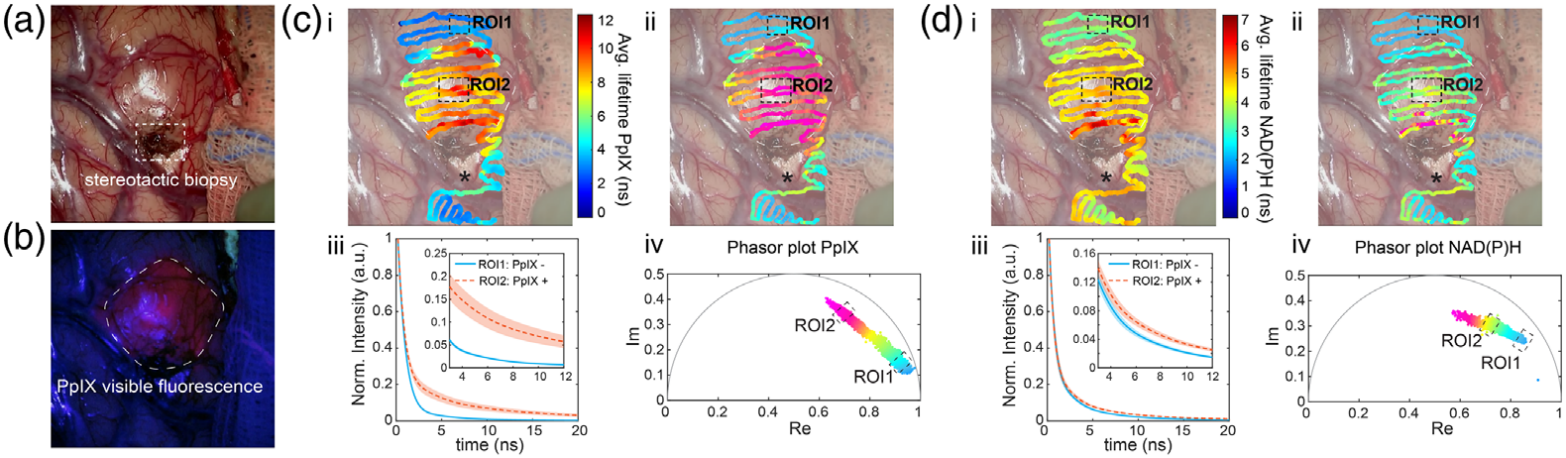
FLIm of a superficial GBM tumor. (a) White light image of the surgical field-of-view (FOV). (b) Standard fluorescence microscope image (seen on the surgical microscope) used for 5-ALA visualization (excitation 405 nm). Augmented fluorescence lifetime of the PpIX channel (629/53 nm) (c) and the NAD(P)H channel (470/28 nm) (d) (i) Average fluorescence lifetime overlayed on the surgical FOV. (ii) Phasor coordinates overlayed on the surgical FOV color-coded according to the phasor histogram in (iv). (iii) Mean fluorescence decay (curve) and standard deviation (shaded area) from regions of interest (ROIs) 1 and 2 representative of PpIX negative and PpIX positive areas, respectively. Inserts show a zoomed in section of the decays. N=300 points per ROI. Videos 1 and 2 show data acquisition and lifetime overlay on the surgical FOV for the PpIX and the NAD(P)H channels, respectively (Video 1, MP4, 26, 967 KB [URL: https://doi.org/10.1117/1.JBO.27.2.020501.1] ; Video 2, MP4, 27,131 KB [URL: https://doi.org/10.1117/1.JBO.27.2.020501.2] ).

Figure 1 shows FLIm data acquired before tumor resection during a craniotomy procedure from a GBM patient presenting a tumor-infiltrated cortex [Fig. 1(a)]. The visible PpIX fluorescence as seen through the surgical microscope is shown in Fig. 1(b). The PpIX positive area exhibited an increased average lifetime on the PpIX channel (629/53 nm) ($\tau_{\mathrm{avg}}^{\mathrm{ROI}2}=9.3\pm 0.9\;\mathrm{ns}$) compared with the surrounding tissue with no visible PpIX fluorescence ($\tau_{\mathrm{avg}}^{\mathrm{ROI}1}=3\pm 0.3\;\mathrm{ns}$) [Fig. 1(ci), Video 1]. The mean decays from the selected regions of interest [Fig. 1(ciii)] further illustrate the long-lasting decay on the PpIX positive area. The corresponding phasors aligned between a fast component (ROI1) and a slow (ROI2) component [Fig. 1(civ)]. The slow component was spatially overlapped with the PpIX positive area [Fig. 1(cii)], whereas distant areas with no visible PpIX fluorescence were characterized by fast decays. The fluorescence lifetime was heterogeneous around the tumor area, with decreasing average lifetime on the borders of the visible fluorescence region [Fig. 1 (ci,ii)]. These results agree with previous studies conducted on *ex vivo* brain samples with a mesoscopic frequency-domain FLIM approach that employed a modulated 405 nm excitation light.^6^^,^^16^ The long-lasting component was attributed to pure PpIX, which has a long fluorescence lifetime (∼16  ns) in solvent, and the fast decay was consistent with non-pathological tissue autofluorescence.

The average lifetime on the NAD(P)H channel also increased in the area with visible PpIX fluorescence although to a lesser extent [$\tau_{\mathrm{avg}}^{\mathrm{ROI}2}=4.84\pm 0.18\;\mathrm{ns}$ versus $\tau_{\mathrm{avg}}^{\mathrm{ROI}1}=3.49\pm 0.17\;\mathrm{ns}$ surrounding tissue; Fig. 1(di), Video 2]. The mean decays show a small but noticeable difference between the two areas [Fig. 1(diii)]. The strongest contrast in NAD(P)H lifetime was located around an area of scar tissue resulting from a stereotactic biopsy performed prior to surgery [Fig. 1(a)], featuring a long average lifetime (>6  ns). This additional information is not conveyed in the PpIX channel. The phasor representation of the NAD(P)H fluorescence also highlighted the PpIX-positive area (ROI2) and the scar tissue with the longest lifetime components [Fig. 1(dii,iv)]. Unaffected tissue (ROI1) featured the fastest decays.

Assuming that the major contribution to 470/28 nm fluorescence comes from NAD(P)H, the fastest components may indicate an abundance of free NAD(P)H (short lifetime ∼0.4  ns^21^) and decreased contribution from bound NAD(P)H (2 to 4 ns depending on the binding protein^21^). An abundance of free NAD(P)H is typically associated with the glycolytic state of many cancer cells. However, recent experiments have challenged this theory for gliomas, including GBM, suggesting a more complex metabolism in which oxidative pathways co-exist with aerobic glycolysis and even implying that different cell types within the tumor employ different metabolic strategies.^22^^,^^23^ These results may explain why the average fluorescence lifetime was not necessarily shorter in GBM tumors. Previous studies have also reported long average lifetimes from GBM tumors compared with surrounding healthy parenchyma from both excised^24^ and *in vivo* tissue.^25^

A region with a distinct lifetime (black asterisk, Fig. 1(c) and Fig. 1(di,ii)] overlayed a cerebral sulcus, with an abundance of blood vessels surrounded by cerebrospinal fluid, and less brain parenchyma. Although distinct in the PpIX channel [Fig. 1(ci)], this area appears indistinguishable from the tumor based only on the NAD(P)H average lifetime [Fig. 1(di)]. The phasor representation, however, indicated different decay dynamics in the region that could be further explored to differentiate tissue types (Fig. S1 in the Supplementary Material).

### 3.2 Glioma Infiltrating the White Matter (Resection Margins)

**Fig. 2:**
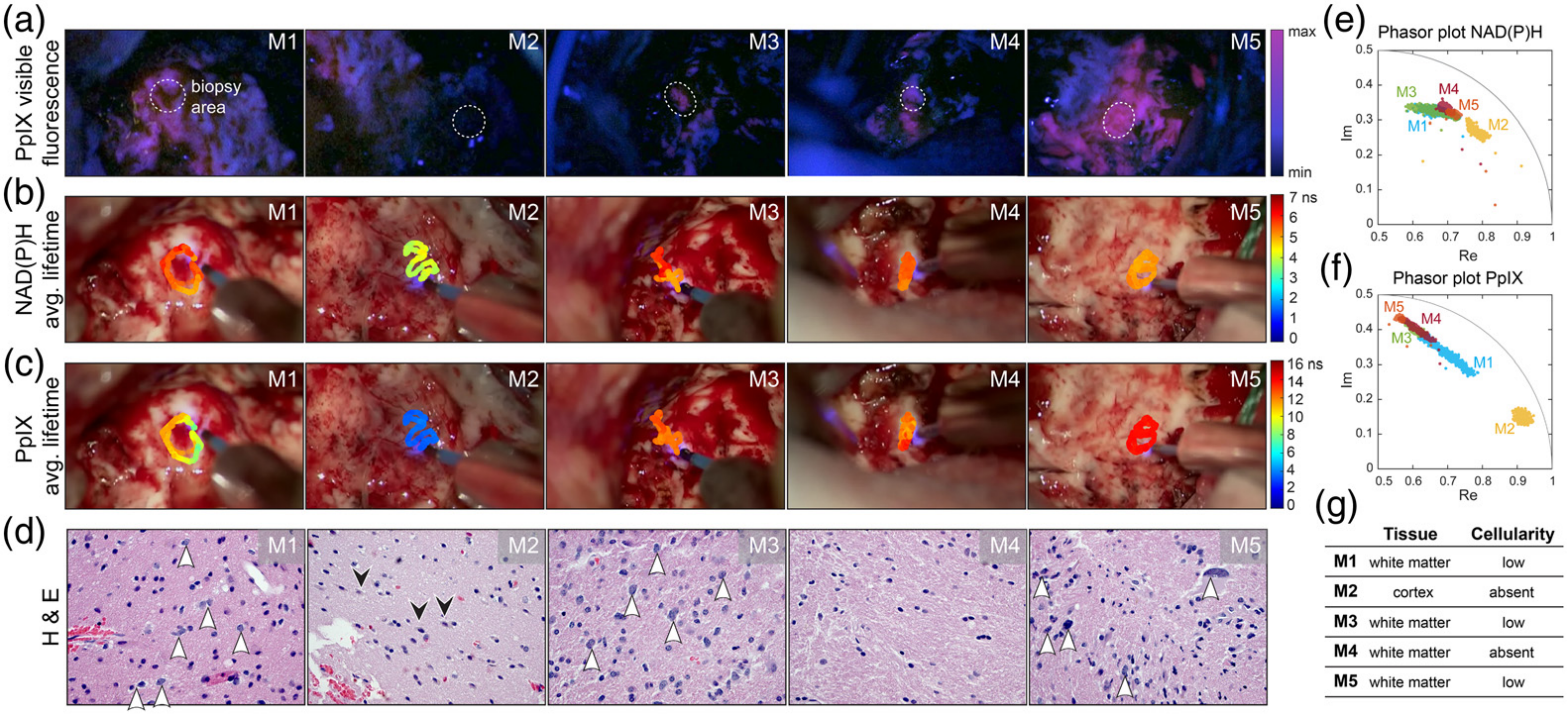
FLIm-based assessment of 5 GBM resection margins (M1 – M5) from one patient. (a) 5-ALA-induced PpIX visible fluorescence images of the surgical FOV as seen with the surgical microscope. The dashed circles indicate the area where FLIm measurements and biopsies were collected. (b) Overlay of the average fluorescence lifetime maps on the surgical FOV for the NAD(P)H channel and (c) PpIX channel. (d) Representative H&E staining images from the corresponding margin at 400× magnification, white arrowheads highlight the tumor cell density of each margin. Black arrowheads in M2 indicate the presence of neurons. (e) Phasor plot representation of the fluorescence decays for each margin in the NAD(P)H channel and (f) PpIX channel. (g) Summary of tissue type and degree of tumor cellularity according to the neuropathology evaluation.

Figure 2 shows five margin areas (M1 – M5) from one representative patient with different levels of PpIX visible fluorescence and tumor cellularity content defined as low or absent by histopathology.

Margins 1 (M1) and M3 were identified as positive for visible PpIX fluorescence [Fig. 2(a)] and contained a low number of tumor cells infiltrating in white matter [white arrowheads, Fig. 2(d)]. These two areas had similar NAD(P)H average lifetimes [$\tau_{\mathrm{avg}}^{M1}=5.5\pm 0.3\;\mathrm{ns}$, $\tau_{\mathrm{avg}}^{M3}=5.5\pm 0.4\;\mathrm{ns}$; Fig. 2(b)] and overlapping phasor clusters [Fig. 2(e)]. The PpIX average lifetime from M1 was shorter than M3 [$\tau_{\mathrm{avg}}^{M1}=9.9\pm 1.2\;\mathrm{ns}$ versus $\tau_{\mathrm{avg}}^{M3}=12.2\pm 0.4\;\mathrm{ns}$; Fig. 2(c)], and it presented a greater variation across the imaged area as illustrated in the phasor cluster [Fig. 2(f)]. Such elongation of the phasor hints at a heterogeneous distribution of PpIX concentration. This distribution could be further explored to develop a quantitative approach for PpIX fluorescence detection.^6^

M2 was collected from a cortex area with no visible PpIX fluorescence [Fig. 2(a)] and the absence of identifiable tumor cells [Fig. 2(d)]. The M2 average fluorescence lifetime was the shortest for this patient in both spectral channels ($\tau_{\mathrm{avg}}^{M2}=4\pm 0.2\;\mathrm{ns}$ for NAD(P)H and $\tau_{\mathrm{avg}}^{M2}=3.5\pm 0.3\;\mathrm{ns}$ for PpIX), as seen in both augmented images [Figs. 2(b) and 2(c)] and phasor plots [Figs. 2(e) and 2(f)]. Previous studies have shown a shorter average lifetime from NAD(P)H on the cortex compared with white matter,^9^ which may also influence the fast decays detected from this margin sample.

M4 was acquired from a white matter area with weak visible PpIX fluorescence [Fig. 2(a)] but with no identifiable tumor cells [Fig. 2(d)]. However, in agreement with the weakly visible PpIX fluorescence, FLIm in the PpIX channel showed a long-lasting emission [$\tau_{\mathrm{avg}}^{M4}=12.1\pm 0.8\;\mathrm{ns}$; Fig. 2(c)], and the corresponding phasor clustered with the rest of the PpIX positive samples [Fig. 2(f)]. This example showcases a limitation of 5-ALA-based FGS. Although the positive predictive value of 5-ALA is extremely high for bulk high-grade gliomas, its performance is known to decrease around the infiltrative edges.^26^ In the NAD(P)H channel, the average lifetime [$\tau_{\mathrm{avg}}^{M4}=5.4\pm 0.2\;\mathrm{ns}$; Fig. 2(b)] was similar to other areas with low tumor cellularity; however, it presented a distinct orientation on the phasor plot [Fig. 2(e)]. With current data, it is unclear what may cause this variation, but this suggests that distinct metabolic activity or biochemical makeup could help discriminate positive tumor areas around the resection margins.

M5 showed the strongest visible PpIX fluorescence of the set [Fig. 2(a)]. Pathology confirmed that there was a low density of tumor cells in this sample but with increased nuclear atypia seen in many of the cells [Fig. 2(d) and Fig. S2 in the Supplementary Material]. The average lifetime in the PpIX channel was the longest measured ($\tau_{\mathrm{avg}}^{M5}=13.7\pm 0.2\;\mathrm{ns}$), and the associated phasor was tightly clustered at the slow end of the phasor plot. On the NAD(P)H channel, this sample exhibited a shorter lifetime than the rest of the margins collected from white matter [$\tau_{\mathrm{avg}}^{M5}=5\pm 0.2\;\mathrm{ns}$; Fig. 2(b)].

**Fig. 3:**
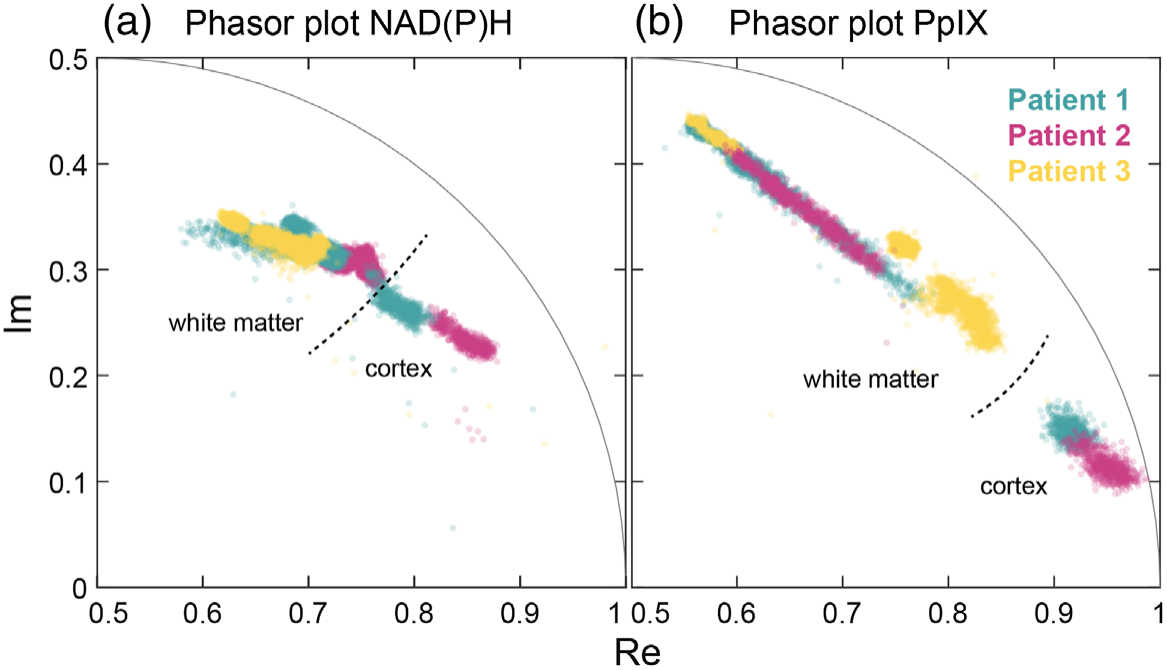
Phasor plot representation of *in vivo* FLIm data in (a) the NAD(P)H and (b) the PpIX channels from areas around the resection margins of 3 GBM patients. Most samples represented here were obtained from white matter, with two obtained from cortical parenchyma.

Figure 3 shows that data acquired from the resection margins of all three GBM patients were aligned and spanned within the same range. Figure 3(a), shows the NAD(P)H phasors inside the universal circle, which hints at multi-exponential decays. It is expected that the extreme value at the fast end (∼Re=0.9, Im = 0.2) points towards the single exponential decay of free NAD(P)H, whereas the opposite ends point toward long-lasting components. Samples collected from other patients align with either one of the different directions observed in Fig. 2 (M1/M3 or M4/M5). A plausible explanation for the multiple slower components is NAD(P)H binding to different proteins. The final position of the phasor cluster would indicate the ratio of free to bound NAD(P)H within a given sample. Nonetheless, the long-lasting decays could also have contributions from other fluorescence species (i.e., flavins, trace collagen, or even lipids from the myelin sheaths) weakly emitting into this spectral band. The phasor distributions of the margin samples also overlapped in the PpIX channel [Fig. 3(b)]. The overall phasor shape indicates a bi-exponential decay with a very short component and a very long component at the extremes. A similar elongation of the phasor plot was observed on the cortex data (Sec. 3.1) and recently on excised brain samples.^6^^,^^16^

In summary, areas with low tumor cellularity presented longer PpIX decays than samples with no tumor cells (e.g., M1, M3, and M5 versus M2). Out of 15 total samples collected, one (M4) appeared positive for PpIX visible fluorescence with an associated long PpIX lifetime but showed no presence of tumor cells as confirmed by histopathology. The relationship between NAD(P)H lifetime and tumor cellularity is more nuanced and complex and needs to factor in the underlying tissue type (i.e., cortex or white matter). Additional studies will determine the tumor margin diagnostic ability of the two lifetime metrics and the relationship between PpIX fluorescence and tissue metabolism.

## 4 Conclusion

We report the first *in vivo* intraoperative FLIm measurements that simultaneously detected 5-ALA-induced PpIX fluorescence and NAD(P)H emission of brain tissue from GBM patients. The current implementation uses a fiber probe for hand-scanning, point-measurement imaging. Although this approach may acquire data more slowly than a wide-field imaging approach for large and flat surfaces,^16^ point scanning allows the surgeon to navigate the complex geometry of resection cavities with increased flexibility. Each point measurement is acquired in <10  μs and augmented on the surgical field-of-view at video rate (33 ms). In this way, large areas (centimeters) can be covered in seconds, improving biopsy frozen section times (∼20  min) and reducing sampling errors. Moreover, the implemented pulse sampling approach enables FLIm measurements under normal illumination, unlike current frequency-domain approaches.^16^ This is a key advantage over wide-field imaging through the microscope that allows rapid, simultaneous scanning and resection, leading to a more efficient process that is less disruptive of the surgical workflow.

The simultaneous and co-registered collection of PpIX and NAD(P)H fluorescence enables a clinical tool to identify tumor areas while performing tumor resection and doubles as a research tool to study tumor environment and metabolism *in vivo*. Each set of signals (PpIX and NAD(P)H), and their combination, forms a set of clinically valuable data that can be used as features for algorithms that predict tumor areas with different degrees of malignancy. The approach may also benefit from additional spectral bands to obtain information from structural proteins, pyridoxines, and flavins for enhanced biochemical and metabolic specificity. This, combined with PpIX fluorescence, may enable more accurate detection of the infiltration zone of high-grade gliomas, including GBM, as well as low-grade gliomas, in which visible 5-ALA fluorescence intensity has poor sensitivity.^4^

Current results demonstrate the ability of intraoperative FLIm to simultaneously detect co-registered PpIX and NAD(P)H fluorescence from the tumor resection margins in an integrated approach with the surgical workflow (i.e., under standard room-light illumination). The presented findings support further studies to establish the relationships between tumor presence and PpIX and NAD(P)H fluorescence lifetime to determine their diagnostic ability. Intraoperative FLIm of 5-ALA-induced PpIX and tissue autofluorescence makes a promising surgical adjunct to guide tumor resection surgery that could have a direct impact on patient outcome.

## Code, Data and Materials Availability

The datasets generated for this study are available upon request to the corresponding author.

## References

[1] Palmieri G. et al., “Fluorescence-guided surgery for high-grade gliomas: state of the art and new perspectives,” Technol. Cancer Res. Treat. 20, 153303382110216 (2021). 10.1177/15330338211021605 PMC8255554 34212784

[2] Stepp H. Stummer W., “5-ALA in the management of malignant glioma,” Laser Surg. Med. 50(5), 399–419 (2018). 10.1002/lsm.22933 29737540

[3] Valentine R. M. et al., “Modelling fluorescence in clinical photodynamic therapy,” Photochem. Photobiol. Sci. 12(1), 203–213 (2012). PPSHCB 1474-905X 10.1039/C2PP25271F 23128146

[4] Kiesel B. et al., “5-ALA in suspected low-grade gliomas: current role, limitations, and new approaches,” Front. Oncol. 11, 699301 (2021). FRTOA7 0071-9676 10.3389/fonc.2021.699301 34395266 PMC8362830

[5] Belykh E. et al., “Optical characterization of neurosurgical operating microscopes: quantitative fluorescence and assessment of PpIX photobleaching,” Sci. Rep.-UK 8(1), 12543 (2018). 10.1038/s41598-018-30247-6 PMC6105612 30135440

[6] Reichert D. et al., “Fluorescence lifetime imaging and spectroscopic co-validation for protoporphyrin IX-guided tumor visualization in neurosurgery,” Front. Oncol. 11, 741303 (2021). FRTOA7 0071-9676 10.3389/fonc.2021.741303 34595120 PMC8476921

[7] Molina E. S. et al., “5-Aminolevulinic acid-induced porphyrin contents in various brain tumors: implications regarding imaging device design and their validation,” Neurosurgery 89, 1132–1140 (2021). NEQUEB 10.1093/neuros/nyab361 34670277

[8] Sun Y. et al., “*In vivo* validation of a bimodal technique combining time-resolved fluorescence spectroscopy and ultrasonic backscatter microscopy for diagnosis of oral carcinoma,” J. Biomed. Opt. 17(11), 116003 (2012). JBOPFO 1083-3668 10.1117/1.JBO.17.11.116003 23117798 PMC3484195

[9] Alfonso-Garcia A. et al., “Real-time augmented reality for delineation of surgical margins during neurosurgery using autofluorescence lifetime contrast,” J. Biophotonics, 13, e201900108 (2019). 10.1002/jbio.201900108 31304655 PMC7510838

[r10] Kantelhardt S. R. et al., “*In vivo* multiphoton tomography and fluorescence lifetime imaging of human brain tumor tissue,” J. Neuro-Oncol. 127(3), 473–482 (2016). JNODD2 0167-594X 10.1007/s11060-016-2062-8 26830089

[11] Lukina M. et al., “Label-free macroscopic fluorescence lifetime imaging of brain tumors,” Front. Oncol. 11, 666059 (2021). FRTOA7 0071-9676 10.3389/fonc.2021.666059 34109119 PMC8181388

[12] Tommasini-Ghelfi S. et al., “Cancer-associated mutation and beyond: the emerging biology of isocitrate dehydrogenases in human disease,” Sci. Adv. 5(5), eaaw4543 (2019). STAMCV 1468-6996 10.1126/sciadv.aaw4543 31131326 PMC6530995

[13] Kim J. E. et al., “Mechanism for enhanced 5-aminolevulinic acid fluorescence in isocitrate dehydrogenase 1 mutant malignant gliomas,” Oncotarget 6(24), 20266–20277 (2015). 10.18632/oncotarget.4060 26008980 PMC4653003

[14] Traylor J. I. et al., “Molecular and metabolic mechanisms underlying selective 5-aminolevulinic acid-induced fluorescence in gliomas,” Cancers 13(3), 580 (2021). 10.3390/cancers13030580 33540759 PMC7867275

[15] Butte P. V. et al., “Fluorescence lifetime spectroscopy for guided therapy of brain tumors,” Neuroimage 54, S125–S135 (2011). NEIMEF 1053-8119 10.1016/j.neuroimage.2010.11.001 21055475 PMC3335732

[16] Erkkilä M. T. et al., “Macroscopic fluorescence-lifetime imaging of NADH and protoporphyrin IX improves the detection and grading of 5-aminolevulinic acid-stained brain tumors,” Sci. Rep.-UK 10(1), 20492 (2020). 10.1038/s41598-020-77268-8 PMC7686506 33235233

[17] Zhou X. et al., “Multispectral fluorescence lifetime imaging device with a silicon avalanche photodetector,” Opt. Express 29(13), 20105 (2021). OPEXFF 1094-4087 10.1364/OE.425632 34266107 PMC8237936

[18] Gorpas D. et al., “Autofluorescence lifetime augmented reality as a means for real-time robotic surgery guidance in human patients,” Sci. Rep.-UK 9(1), 1187 (2019). 10.1038/s41598-018-37237-8 PMC6362025 30718542

[19] Liu J. et al., “A novel method for fast and robust estimation of fluorescence decay dynamics using constrained least-squares deconvolution with Laguerre expansion,” Phys. Med. Biol. 57(4), 843–865 (2012). PHMBA7 0031-9155 10.1088/0031-9155/57/4/843 22290334 PMC3407553

[20] Digman M. A. et al., “The phasor approach to fluorescence lifetime imaging analysis,” Biophys. J. 94(2), L14–L16 (2008). BIOJAU 0006-3495 10.1529/biophysj.107.120154 17981902 PMC2157251

[21] Lakowicz J. R., Principles of Fluorescence Spectroscopy, Springer, New York (2006).

[22] Lin H. et al., “Fatty acid oxidation is required for the respiration and proliferation of malignant glioma cells,” Neuro-Oncology 19(1), 43–54 (2017). 10.1093/neuonc/now128 27365097 PMC5193020

[23] Strickland M. Stoll E. A., “Metabolic reprogramming in glioma,” Front. Cell Dev. Biol. 5, 43 (2017). 10.3389/fcell.2017.00043 28491867 PMC5405080

[24] Marcu L. et al., “Fluorescence lifetime spectroscopy of glioblastoma multiforme,” Photochem. Photobiol. 80(1), 98–103 (2004). PHCBAP 0031-8655 10.1562/2003-12-09-RA-023.1 15339216

[25] Sun Y. et al., “Fluorescence lifetime imaging microscopy for brain tumor image-guided surgery,” J. Biomed. Opt. 15(5), 056022 (2010). JBOPFO 1083-3668 10.1117/1.3486612 21054116 PMC2966493

[26] Valle R. D. Hadjipanayis C. G. Stummer W., “Established and emerging uses of 5-ALA in the brain: an overview,” J. Neuro-Oncol. 141(3), 487–494 (2019). JNODD2 0167-594X 10.1007/s11060-018-03087-7 30607705

