# Supplementary material for: First in patient assessment of brain tumor infiltrative margins using simultaneous time-resolved measurements of 5-ALA-induced PpIX fluorescence and tissue autofluorescence
Source: J Biomed Opt. 2022 Feb 2;27(2):020501. doi: 10.1117/1.JBO.27.2.020501 (PMC8809358; doi:10.1117/1.JBO.27.2.020501)
Supplement: Supplementary file 1 [file JBO_027_020501_SD001.pdf]

First in patient assessment of brain tumor infiltrative margins using simultaneous time-resolved measurements of 5-ALA-induced PpIX fluorescence and tissue autofluorescence

Alba Alfonso-García,[a] Xiangnan Zhou,[a] Julien Bec,[a] Silvia Noble Anbunesan,[a] Farzad Fereidouni,[a,b] Lee-Way Jin,[b] Han Sung Lee,[b] Orin Bloch,[c] Laura Marcu[a,c,*]
[a]University of California Davis, Department of Biomedical Engineering, Davis, US
[b]University of California Davis, Department of Pathology and Laboratory Medicine, Sacramento, US
[c]University of California Davis, Department of Neurological Surgery, Sacramento, US

Supplemental Material

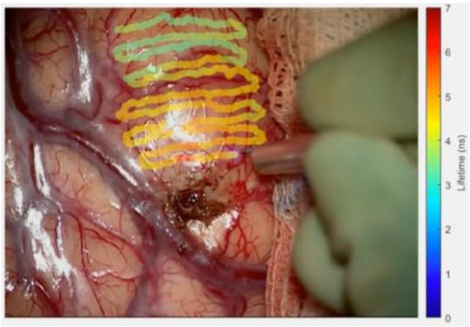

Video 1: Average fluorescence lifetime acquired at 470/28 nm (NAD(P)H channel) augmented on the surgical FOV of a superficial GBM tumor infiltrating the cortex. The neurosurgeon scans the cortex surface with the optical fiber probe to record FLIm data in real time.

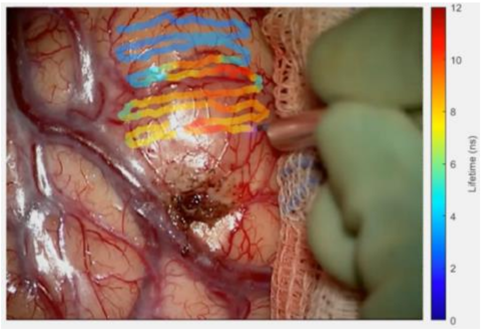

Video 2: Average fluorescence lifetime acquired at 629/53 nm (PpIX channel) augmented on the surgical FOV of a superficial GBM tumor infiltrating the cortex. The neurosurgeon scans the cortex surface with the optical fiber probe to record FLIm data in real time.

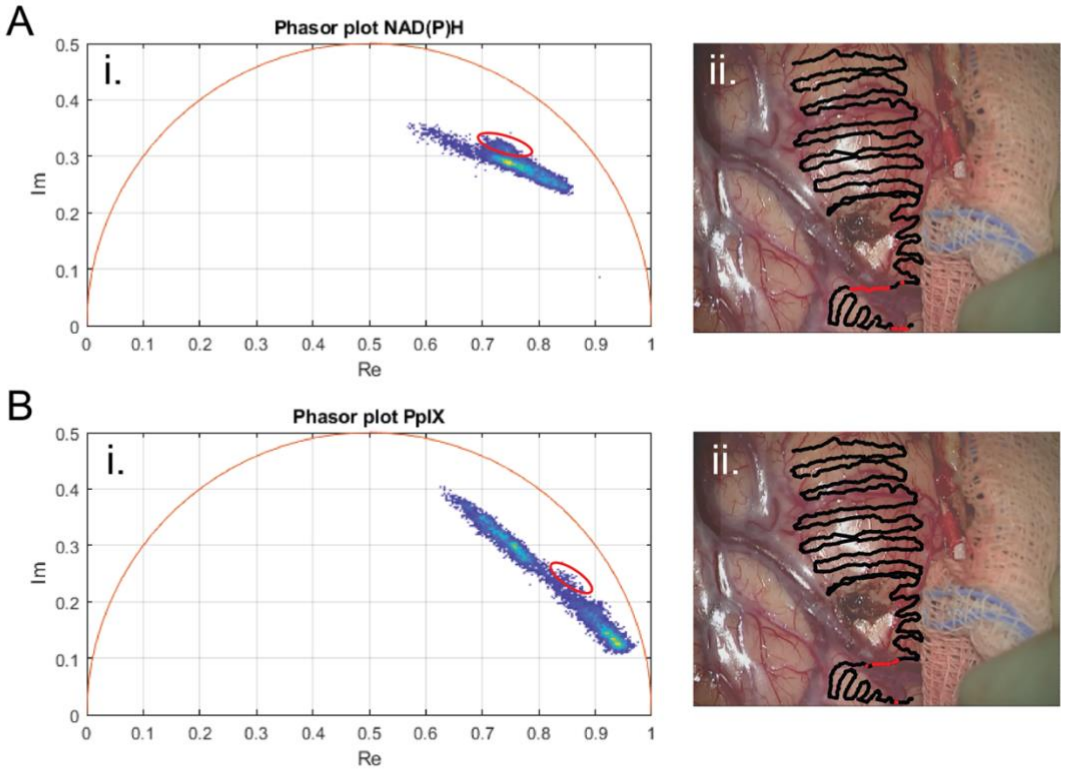

Fig. S1 Phasor plot histogram for fluorescence decays in the NAD(P)H (A) and the PpIX (B) channels. The red circle outlines the phasor coordinates (i) from the points highlighted in red on the overlay with the surgical FOV (ii). This portion of phasors, which cluster in a slightly different direction than the bulk of the coordinates in the NAD(P)H channel and on a side lobe in the PpIX channel, spatially overlaps a sulcus between 2 regions of normal brain tissue.

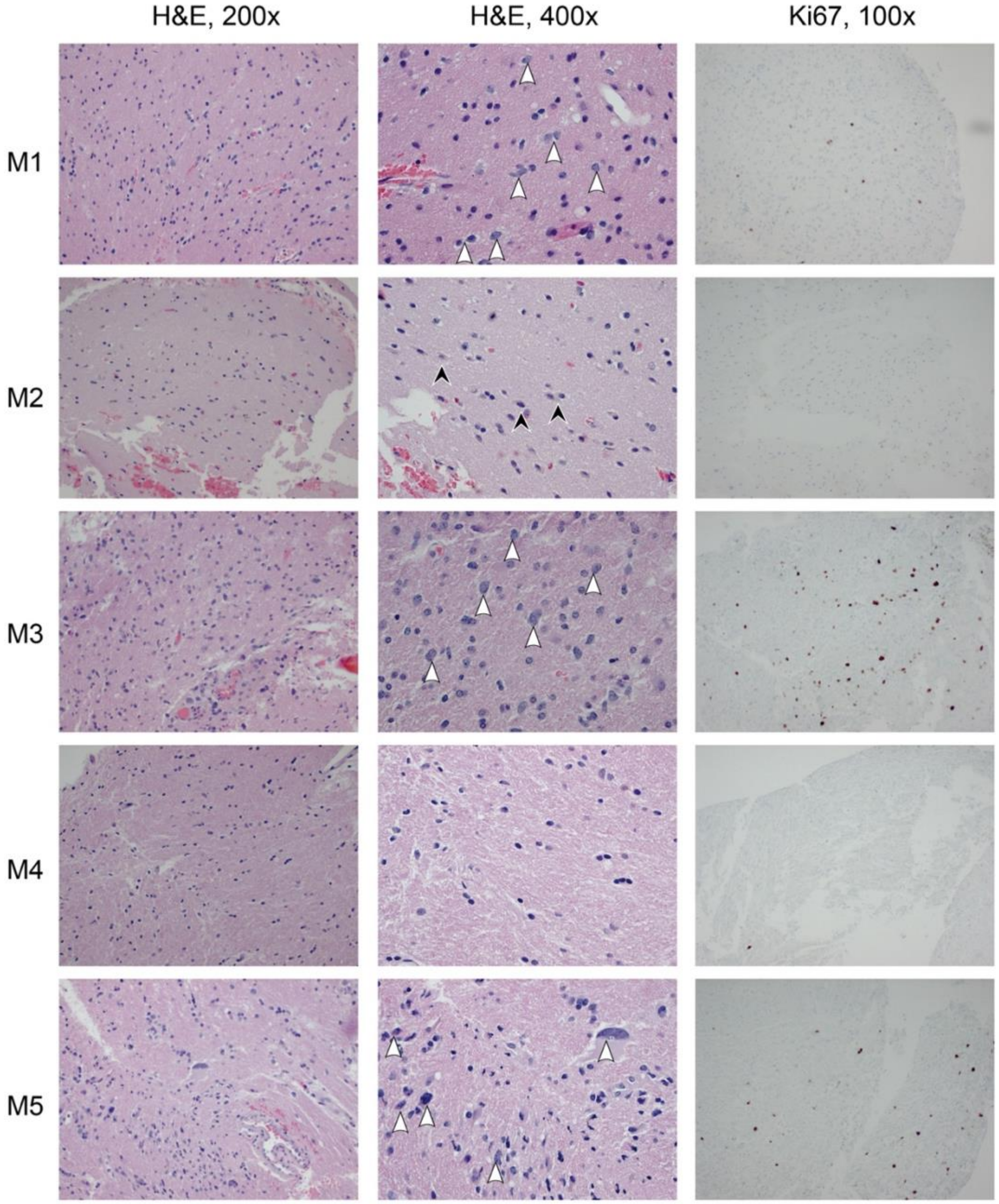

Fig. S2 Pathology of margins. Images of H&E stained tissue from margins M1-M5 shown at medium (200x,) and high (400x, same as Fig. 2) magnifications and of Ki67 immunohistochemistry of the respective margins at lower magnification (100x) show the overall cellularity, the presence of glial tumor cells (white arrowheads pointing a subset of tumor cells) and the presence of neurons in cortex (black arrowheads point to subset of neurons). Multiple Ki67 positively stained nuclei (brown spots) are present in M1, M3 and M5, demonstrating the actively proliferating population of tumor cells in those margins.
